# 

**Published:** 1889-05-11

**Authors:** 


					PRICS ONE IPENNY.;
No. J37, Vol. VI.
-May if 1th, -1889^
Per Annum, 6s. 6d., post free.
Monthly Parts,' 6d.; jost free, 7?d,
Ditto per Annum, 7s. 6d., post free.
i-?.wcgi3i,ereu. at me uenerai rose umce as a i^ewsyu^ *..j

				

